# Preventive Medication Use among Adults Aged 40 and over in the United States: National Health and Nutrition Examination Survey, 2015–2018

**DOI:** 10.3390/healthcare10101875

**Published:** 2022-09-26

**Authors:** Abdulkarim M. Meraya

**Affiliations:** Department of Pharmacy Practice, Faculty of Pharmacy, Jazan University, Jazan P.O. Box 114-45124, Saudi Arabia; ameraya@jazanu.edu.sa

**Keywords:** preventive, prescription medications, chronic diseases, NHANES, aspirin

## Abstract

Objectives: 1. To estimate the prevalence of preventive medication use among adults in the United States (US); 2. To identify the socioeconomic, demographic and clinical factors associated with preventive prescription medication use; 3. To identify the diagnoses associated with preventive prescription medication use. Methods: Data from two cycles of the National Health and Nutrition Examination Survey, 2015–2016 and 2017–2018, are analyzed. Results: Among US adults aged 40 years or older (N = 7634), 31% use a preventive medication. Specifically, 27% of them use aspirin and 9% use other preventive prescription medications. Among those who use other preventive prescription medications, 27% report using one of the cardiovascular agents and 24% reported using anticoagulants and/or antiplatelet agents. High percentages of preventive prescription medication users report using medications to prevent heart attacks/myocardial infarctions (25%) or blood clots (23%). Uninsured adults are less likely to use preventive medications (OR: 0.656; *p*-value = 0.009) as compared with their counterparts with private insurance. On the other hand, older adults and those with diabetes, heart disease, arthritis and hypertension are more likely to use preventive medications. Furthermore, past smokers and adults who never smoked are more likely to use preventive medications as compared with those who currently smoke. Conclusion: Policies are needed to increase access to preventive care for uninsured adults. Further research needs to review the benefits and harms of the chronic use of preventive medication among older adults in the US.

## 1. Introduction

Preventive pharmacotherapy is widely used in the United States (US) [1,2,3]. Prescribers in the US use preventive medications either as primary prevention strategies to prevent chronic physical conditions before they occur, or as secondary prevention strategies to prevent complications from existing chronic physical conditions [1,2,3]. Common preventive medications including aspirin and lipid-lowering agents are among the most prescribed medications in the US [1,2,3,4]. These medications are used to prevent the occurrence or the complications of cardiovascular diseases in individuals at high-risk or who currently have the disease [2]. Preventive medications are not only used to prevent cardiovascular disease, but also to prevent migraines, cancer, type 2 diabetes mellitus, osteoporosis, gout and others [5,6,7,8].

In 2012, 52% of US adults aged 45–75 years reported the use of aspirin for the primary or secondary prevention of cardiovascular disease [3]. From 2011–2012, 28% of US adults aged 40 and over reported using lipid-lowering medications, and the percentage increased among individuals with cardiovascular disease (70%) or diabetes (63%) [1]. Antiplatelets, glucose-lowering drugs, antimigraine drugs and selective estrogen receptor modulators are also widely used as preventive therapies in the US [4,5,6,7,8]. In the US, previous studies have focused on the preventive medications for cardiovascular disease; however, a comprehensive review of the prevalence of preventive prescription medication use is lacking [2]. Moreover, little is known about the socioeconomic and demographic factors associated with preventive medication use.

Therefore, this study had three objectives: 1. To estimate the prevalence of preventive medication use among adults in the US; 2. To identify the socioeconomic, demographic and clinical factors associated with preventive medication use; and 3. To identify the diagnoses associated with preventive prescription medication use.

## 2. Materials and Methods

This is a retrospective cross-sectional study using data from two cycles of the National Health and Nutrition Examination Survey (NHANES), 2015–2016 and 2017–2018. The NHANES is conducted by US National Center for Health Statistics, Centers for Disease Control and Prevention to provide nationally representative statistics for the US population. The NHANES provides information on the health and nutritional statuses of children and adults in the US. In this study, demographic data and data on medical conditions and prescription medications were used to estimate the prevalence of preventive medication use among adults. The data used in this study are publicly available at https://www.cdc.gov/nchs/nhanes/ (accessed on 5 February 2022).

### 2.1. Study Sample

The study sample consisted of adults aged 40 years or older with no missing data on preventive medication use (N = 7634). The total number of individuals in the selected two NHANES cycles was 19,225. Of those individuals, 11,577 were excluded as they were <40 years old at the time of screening. Fourteen individuals were excluded because of missing information on preventive medications use.

### 2.2. Measurements

#### 2.2.1. Outcome

The primary outcome was to determine the level of preventive medication use among adults in the US. The NHANES gives information on the prescription medication use reported by survey participants. The participants were asked “In the past 30 days, have you used or taken medication for which a prescription is needed?” The NHANES also provides information on the generic name of the drug, the duration of the use in days and the reasons for the use. Participants also reported whether they use low-dose preventive aspirin following medical advice or on their own. Preventive medication users were defined as those who reported using any medication to prevent any disease in the past 30 days or reported using low-dose preventive aspirin. In the analysis, this study did not differentiate between the acute use and chronic use of preventive medications. Furthermore, this study did not differentiate between primary prevention vs secondary prevention for diseases.

#### 2.2.2. Explanatory Variables

The explanatory variables included demographic variables, chronic physical conditions and depression. The demographic variables were sex, age, race–ethnicity, marital status, education level, poverty, health insurance and smoking status. Age was categorized to four levels: 40–49, 50–64, 65–74 and ≥75 years. Race–ethnicity was categorized to Mexican Americans, other Hispanic, non-Hispanic white, non-Hispanic black and other. The marital status categories were married, living with partner, widowed, divorced, separated and never married. The poverty levels in the study were categorized as poor (poverty-ratio index < 100%) and non-poor. Health insurance levels were categorized as uninsured, private only, public only, private and public insurance and other. Smoking status was categorized as current smokers, past smokers and never smokers. Chronic physical conditions included arthritis (yes, no), diabetes (yes, no), heart disease (yes, no) and hypertension (yes, no). Other chronic physical conditions were asthma, emphysema, thyroid problems, chronic bronchitis, chronic obstructive pulmonary disease and cancer. The number of other chronic physical conditions was included in the analyses (no other chronic physical conditions and ≥1 chronic physical conditions). Based on the scores of the Patient Health Quationnaire-9 (PHQ-9) scale, adults in the sample were categorized as adults with depression (score of 10 or greater) and adults without depression (score of less than 10). PHQ-9 is a widely used survey to measure depression, and it is based on nine items that generate scores from 0–27 [9].

### 2.3. Missing Data

The missing data for all of the explanatory variables, with the exclusion of depression, was 1.78% (N missing = 136), and 14% with the inclusion of depression (N missing = 1072). Missing data were associated with age, race, depression and heart disease. Separate analyses were run with depression and without depression as a sensitivity analysis. These analyses found no difference in the results.

### 2.4. Statistical Techniques

Weighted descriptive statistics were computed to calculate survey-weighted estimates of demographic characteristics and comorbidities. The NHANES cycle weights, survey strata and clusters were used to account for oversampling and non-response rates. The survey weights were divided by 2, as two cycles of the NHANES were merged. Chi-square tests were conducted to examine the associations between the explanatory variables and preventive medication use in the bivariate analysis. Since the main outcome of this study was binary (yes, no), multivariable logistic regression models were employed to examine the adjusted relationships between the explanatory variables and preventive medication use. Spearman correlation analysis showed very weak statistically significant correlation coefficients (ranging from −0.0024 to −0.29) between the explanatory variables, suggesting that there was no multicollinearity. The F-adjusted mean residual goodness-of-fit test for logistic regression estimated with survey sample data [10] was applied to assess the model fit. All analyses were conducted using survey procedures in STATA 16.0 (StataCorp, College Station, TX, USA).

## 3. Results

### 3.1. Description of the Study Sample

Table 1 shows the sample characteristics of the study sample. A total of 7634 individuals (weighted N = 150,830,186) aged 40 years or older are included in the present analysis. Of those, 31% use a preventive medication. Further, 53% of the sample are female, 68% are non-Hispanic White and 62% are married.

Preventive medication use is significantly associated with sex, age, race, marital status, type of health insurance, diabetes, arthritis, hypertension, heart disease, presence of other chronic physical conditions, depression and smoking status in the bivariate analysis. Higher proportions of preventive medications users are aged 75 or older than non-preventive medications users (26% vs. 7%). Furthermore, higher proportions of preventive medications users have diabetes (33% vs. 9%), arthritis (55% vs. 33%), hypertension (67% vs. 33%), heart disease (34% vs. 5%) and depression (9% vs. 7%) than those who are non-preventive medications users.

### 3.2. Explanatory Variables and Preventive Prescription Medications Use

The F-adjusted mean residual goodness-of-fit test was applied and suggests no evidence of lack-of-fit of the logistic regression model (adjusted F = 1.336, df = 9, 22, *p*-value = 0.275). The adjusted odds ratios and their 95% confidence interval (CI) for the explanatory variables on preventive prescription medications are displayed in Table 2. In the adjusted analyses, adults between 65–74 years old are significantly more likely to use preventive medications as compared with those 40–49 years of age. Similarly, adults aged 75 years or over are more likely to use preventive medications than those between 40–49 years old. Additionally, females are less likely than males to use preventive medications. Conversely, there is no relationship between race and preventive medication use found in the adjusted analyses.

Uninsured adults aged 40 years or older are less likely to use preventive prescription medications compared with their counterparts with private insurance. Additionally, past smokers and never smokers are more likely to use preventive medications than currently smokers.

All chronic physical conditions are significantly and positively associated with preventive prescription medication use. Adults with diabetes are more likely to use preventive medications than those without. Similarly, adults with heart disease are more likely to use preventive prescription medications than those without. Additionally, those with arthritis, hypertension and a number of other chronic physical conditions are also more likely to use preventive medications.

### 3.3. Preventive Prescription Medications

**Figure 1:**
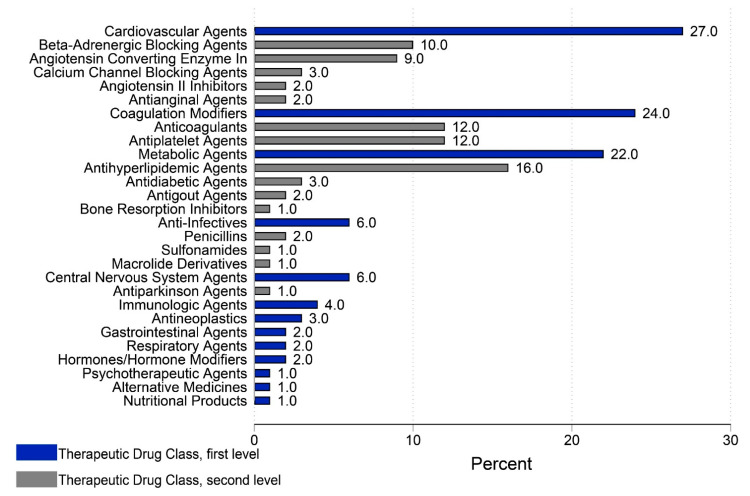
Weighted percentages of prescription medications by therapeutic medication class (N: 1072).

Figure 1 shows the weighted percentages of prescription medications (other than aspirin use) by therapeutic medication class. From 2015–2018, 27% of the preventive prescription medication users reported using one of the cardiovascular agents as a prevention therapy. Specifically, 10% of them reported using beta-adrenergic-blocking agents and 9% reported using angiotensin-converting enzyme inhibitors. Additionally, 24% reported using coagulation modifiers (anticoagulants and/or antiplatelet agents). Furthermore, 22% reported using metabolic agents, including antihyperlipidemic agents (16%). Of the preventive prescription medications users, 6% used anti-infectives to prevent bacterial infection.

### 3.4. Primary Reasons for Using Preventive Prescription Medications

**Figure 2:**
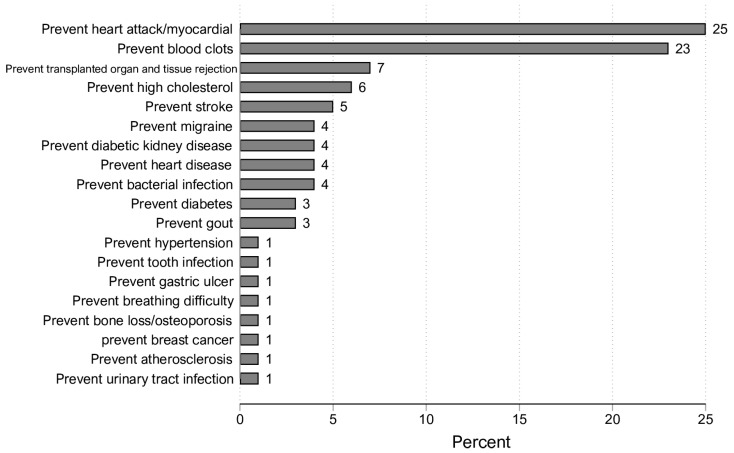
Weighted percentages of reported primary reasons for using preventive prescription medications (N: 995).

Figure 2 lists the most-reported primary reasons for using preventive prescription medications. One quarter of the preventive prescription medication users reported preventing heart attacks/myocardial infarctions the primary reason for using preventive medication therapy. Furthermore, 23% of them reported preventing blood clots as the primary reason for preventive medication therapy.

## 4. Discussion

This study examines the associations between demographic, socioeconomic and clinical factors and preventive prescription medication use among adults aged 40 years or older in the US. In the adjusted analyses, older age and the presence of chronic physical conditions are associated with the use of preventive prescription medications. These results suggest that preventive prescription medications are mainly used as a secondary prevention strategy to prevent complications of existing chronic physical conditions.

The results also suggest that uninsured adults have lower access to medications. In 2015, adults without health insurance were less likely to use preventive aspirin and/or other antiplatelets medications in the US [4]. In 2019, uninsured adults were more likely to not receive prescription medications due to cost as compared with their counterparts with private or public insurance [11]. Furthermore, uninsured adults with chronic conditions were less likely to receive the recommended follow-up services, including preventive care. Preventive care, including preventive prescription medications and follow-up care, is important to prevent disease-related complications and improve the physical and mental health of adults with chronic conditions. Furthermore, Ito et al. found that increasing access to preventive medications, including β-blockers, angiotensin-converting enzyme inhibitors, angiotensin receptor blockers, statins and aspirin, to post-myocardial infarction patients improves health outcomes and also decreases healthcare spending over the long-term [12]. Therefore, policies and programs are required to improve access to preventive care among uninsured adults, especially among those with chronic physical complications.

The results show that older adults are more likely to use preventive medications. Preventive medication use is an effective strategy to prevent disease-related complications, as well as to stop the progress of chronic physical conditions. However, further research is required to determine the benefits and harms of preventive medication use, especially among older adults. For example, aspirin use to prevent cardiovascular disease was deemed helpful for a long time [13]. Nevertheless, the US Preventive Services Task Force (USPSTF) concluded that the net benefit of using preventive aspirin for adults aged 40 to 59 years is small [14]. Furthermore, the USPSTF recommends against initiating aspirin use for the primary prevention of cardiovascular disease in adults 60 years of age or older [14]. Additionally, older adults usually use a high number of long-term medications [15]. Unfortunately, older adults are often excluded from randomized clinical trials. As a result, the benefits and drawbacks of the chronic use of preventive prescription medications are unclear. Therefore, it has been recommended that prescribers should pay more attention and minimize the number of medications among older adults with multimorbid conditions [15].

The foremost consideration for the appropriate use of drugs in a preventive manner would be the strict adherence to the recent changes in clinical guidelines. For example, from 2011–2018, the prevalence of aspirin use among older US adults (≥60 years) was high among individuals at an increased risk for bleeding and other adverse events [16], despite recent changes in the guidelines of the American Heart Association, American Diabetes Association and USPSTF. Moreover, other considerations should be considered before the initiation or continuation of preventive medications use, such as age, multimorbidity, number of other medications and updated guidelines. Furthermore, healthcare professionals need to discuss the benefits and harms of preventive medication use with their patients. Finally, a comprehensive review of preventive medication use in the US is required to determine the net benefits of the use and to determine the consistency of the use with current guidelines.

In this study, current smokers were less likely to use preventive medications. This finding is consistent with previous reports in which smoking status is a risk factor for not receiving eye care and other preventive practices among adults with diabetes [17,18,19]. Smoking is a risk factor of multiple chronic conditions and their related complications. Therefore, it is crucial to understand the low prevalence of preventive practices among smoking adults. Furthermore, it is required to increase the rate of preventive health practices among smoking adults.

This study has many advantages. First, to the best of the author’s knowledge this is the first study to comprehensively review the preventive prescription medication use among adults in the US. Second, this study includes a comprehensive list of clinical, socioeconomic and demographic factors that may affect preventive medications use. Finally, a nationally representative sample of adults aged 40 years or above was included to estimate the prevalence of preventive prescription medication use. Nevertheless, this study has some limitations. This study did not differentiate between chronic and acute preventive medication use. Furthermore, other over-the-counter and non-prescription preventive medications (excluding aspirin) were not included in the analyses of this study.

## 5. Conclusions

More than one-third of adults aged 40 years or older used a preventive medication from 2015–2018 in the US. The majority of the study sample used low-dose preventive aspirin. Uninsured adults were less likely to use preventive medications. Nevertheless, older adults and those with chronic physical conditions were more likely to use preventive prescription medications. Strategies are needed to increase the access to preventive care for uninsured adults. Further research needs to review the preventive prescription medications used in the US.

## Figures and Tables

**Table 1:** Characteristics of the study sample’s preventive prescription medication use (N = 7634). Weighted percentages, National Health and Nutrition Examination Survey, 2015–2016, 2017–2018.

			Aspirin Use and/or Other Preventive Medications			Aspirin Use Only			Other Preventive Medications		
		All	No	Yes	*p*-Value	No	Yes	*p*-Value	No	Yes	*p*-Value
**ALL**			69	31		73	27		91	9	
**Sex**					<0.001			<0.001			0.021
	Female	53	54.8	48.3		54.9	47.2		53.5	46.1	
	Male	47	45.2	51.7		45.1	52.8		46.5	53.9	
**Age groups**					<0.001			<0.001			<0.001
	40–49	26	34.4	7.8		33.3	7		27.9	9.6	
	50–64	42	44.2	36.3		43.6	36.7		42.6	33	
	65–74	19	14.4	29.8		14.9	30.7		18.2	28.9	
	≥75	13	7	26		8.2	25.6		11.4	28.5	
**Race**					<0.001			<0.001			0.034
	Mexican American	7	7.7	5.3		7.6	5.2		7.1	5.9	
	Other Hispanic	6	6	4.6		6	4.3		5.6	5.3	
	Non-Hispanic White	68	65.9	71.4		66	71.9		67.1	72.3	
	Non-Hispanic Black	11	10.7	10.2		10.7	10.1		10.7	8.6	
	Other Race	9	9.7	8.6		9.7	8.5		9.5	8	
**Marital Status**					<0.001			<0.001			0.010
	Married	62	62.8	61.2		62.5	61.9		62.3	63.3	
	Widowed	9	7	13.7		7.4	13.5		8.6	14.1	
	Divorced	14	14.3	12.3		14.3	12.1		14	10.6	
	Separated	3	2.7	2.6		2.7	2.6		2.7	2.4	
	Never Married	7	7.3	6.3		7.4	6		7	6.8	
	Living with Partner	5	5.9	3.9		5.8	3.9		5.5	2.7	
**Education**					0.042			0.064			0.165
	<High School	13	13.2	14.1		13.5	13.3		13.2	15.9	
	High School	24	22.8	25.9		22.7	26.7		23.9	22.3	
	Some College	31	30.6	31.3		31	30.4		30.5	34	
	College +	32	33.4	28.6		32.8	29.6		32.3	27.8	
**Poverty status**					0.367			0.224			0.830
	Poor	11	11.9	10.6		12	9.9		11.4	12.3	
	Near-Poor	18	17.9	19.9		17.9	20.1		18.4	19	
	Middle Income	15	14.8	15.2		15	14.7		14.9	15.4	
	High Income	55	55.5	54.4		55.1	55.3		55.3	53.2	
**Health insurance**					<0.001			<0.001			<0.001
	Private Only	45	52.8	27.9		51.3	28.1		47	25.3	
	Public Only	21	17.1	30.3		18	29.6		19.9	34	
	Public and Private	17.2	11.1	30.9		12.2	30.9		15.7	32.7	
	Other	7.5	7.9	6.6		7.7	6.8		7.8	4.3	
	Uninsured	9	11.2	4.3		10.8	4.5		9.6	3.6	
**Diabetes**					<0.001			<0.001			<0.001
	No	84	90.9	67.3		89.4	67.8		85.8	61.5	
	Yes	16	9.1	32.7		10.6	32.2		14.2	38.5	
**Arthritis**					<0.001			<0.001			<0.001
	No	61	67.4	45.3		66	46.1		62.5	41.3	
	Yes	39	32.6	54.7		34	53.9		37.5	58.7	
**Hypertension**					<0.001			<0.001			<0.001
	No	56	67	32.7		65.1	32.6		58.9	30.9	
	Yes	44	33	67.3		34.9	67.4		41.1	69.1	
**Heart Disease**					<0.001			<0.001			<0.001
	No	86	95.2	66.4		93.7	66.3		89.3	55.3	
	Yes	14	4.8	33.6		6.3	33.7		10.7	44.7	
**Other Chronic Physical Conditions**					<0.001			<0.001			<0.001
	None	59	64	46.7		62.7	47.8		60.3	41.7	
	≥1	41	36	53.3		37.3	52.2		39.7	58.3	
**Depression**					0.012			0.128			0.005
	No	92	92.9	90.6		92.6	91.1		92.6	87.7	
	Yes	8	7.1	9.4		7.4	8.9		7.4	12.3	
**Smoking Status**					<0.001			<0.001			0.052
	Never Smoker	54	55.8	50.2		55.7	49.7		54.2	53.2	
	Currently Smoker	16	17.5	12.4		17	12.8		16.3	11.8	
	Past Smoker	30	26.7	37.4		27.3	37.5		29.5	35	

**Wt.:** weighted. **Note:** based on 7634 adults aged 40 years or over. The *p*-values were derived from chi-square tests between preventive medication users and non-users. Other chronic physical conditions include asthma, emphysema, thyroid problems, chronic bronchitis, chronic obstructive pulmonary disease and cancer.

**Table 2:** Adjusted odds ratios (AOR) and their 95% confidence intervals (CI) of the explanatory variables from multivariable logistic regression on preventive prescription medication use, National Health and Nutrition Examination Survey, 2015–2016, 2017–2018.

		AOR	95% CI	*p*-Value
**Sex**				
	Male	Reference (1)		
	Female	0.746	(0.628–0.885)	0.001
**Age Groups**				
	40–49	Reference (1)		
	50–64	2.616	(1.947–3.514)	<0.001
	65–74	4.271	(2.869–6.358)	<0.001
	≥75	6.985	(4.632–10.532)	<0.001
**Race–Ethnicity**				
	Non-Hispanic White	Reference (1)		
	Mexican Americans	0.995	(0.777–1.274)	0.966
	Other Hispanic	0.882	(0.65–1.197)	0.408
	Non-Hispanic Black	0.918	(0.747–1.127)	0.4
	Other Race	0.843	(0.678–1.048)	0.12
**Marital Status**				
	Married	Reference (1)		
	Widowed	0.648	(0.484–0.868)	0.005
	Divorced	0.771	(0.585–1.015)	0.063
	Separated	1.131	(0.63–2.029)	0.671
	Never Married	1.083	(0.612–1.919)	0.777
	Living with Partner	0.674	(0.494–0.92)	0.015
**Health Insurance**				
	Private Only	Reference (1)		
	Public Only	1.075	(0.846–1.365)	0.542
	Public and Private	1.393	(0.946–2.052)	0.091
	Other	1.182	(0.906–1.543)	0.21
	Uninsured	0.656	(0.483–0.892)	0.009
**Smoking Status**				
	Currently Smoker	Reference (1)		
	Past Smoker	1.431	(1.065–1.922)	0.019
	Never Smoker	1.423	(1.053–1.922)	0.023
**Diabetes**				
	No	Reference (1)		
	Yes	2.779	(2.097–3.684)	<0.001
**Arthritis**				
	No	Reference (1)		
	Yes	1.344	(1.052–1.717)	0.020
**Hypertension**				
	No	Reference (1)		
	Yes	2.294	(1.931–2.726)	<0.001
**Heart Disease**				
	No	Reference (1)		
	Yes	5.504	(4.388–6.905)	<0.001
**Other Chronic Physical Conditions**				
	None	Reference (1)		
	≥1	1.188	(0.988–1.429)	0.067

**AOR:** adjusted odds ratio. **Note:** based on 7634 adults aged 40 years or over. Other chronic physical conditions include asthma, emphysema, thyroid problems, chronic bronchitis, chronic obstructive pulmonary disease and cancer.

## Data Availability

This study was based on a publicly available dataset, NHANES, and can be obtained directly from https://www.cdc.gov/nchs/nhanes/ (accessed on 5 February 2022).
